# Riboflavin Deficiency Is Highly Prevalent in Females and Children across High and Low/Middle Income Countries Worldwide

**DOI:** 10.1016/j.tjnut.2025.101277

**Published:** 2026-02-23

**Authors:** Liadhan McAnena, Mary Ward, Adrian McCann, Kristina Pentieva, Leane Hoey, Ryan Barlow, Harry R Jarrett, Maeve A Kerr, JJ Strain, Catherine Hughes, Albert Flynn, Janette Walton, Yvonne Lamers, Parveen Bhatti, Crystal D Karakochuk, Kyly C Whitfield, Michelle Murphy, Pere Cavallé-Busquets, Lorna J Cox, Ann Prentice, Damon A Parkington, Tabasum Makhdoomi, Sengchanh Kounnavong, Guy-Marino Hinnouho, Nelly Birungi, Tim J Green, Helene McNulty

**Affiliations:** 1 Nutrition Innovation Centre for Food and Health, Ulster University, Coleraine, Northern Ireland, United Kingdom; 2 Biomarkers and Nutrition, Bevital AS, Bergen, Norway; 3 School of Food and Nutritional Sciences, University College Cork, Cork, Ireland; 4 Department of Biological Sciences, Munster Technological University, Cork, Ireland; 5 Food, Nutrition and Health Program, Faculty of Land and Food Systems, University of British Columbia, Vancouver, BC, Canada; 6 British Columbia Cancer Research Institute, University of British Columbia, Vancouver, BC, Canada; 7 Department of Applied Human Nutrition, Mount Saint Vincent University, Halifax, NS, Canada; 8 Unit of Preventive Medicine & Biostatistics, Faculty of Medicine & Health Sciences, IISPV, Universitat Rovira i Virgili, Tarragona, Spain; 9 CIBERObn (Centre de Investigación Biomédica en Red, Obesity and Nutrition Branch), ISCIII, Madrid, Spain; 10 Unit of Obstetrics & Gynecology, University Hospital Sant Joan Reus, IISPV, Reus, Spain; 11 Medical Research Council (MRC) Human Nutrition Research, Elsie Widdowson Laboratory, Cambridge, United Kingdom; 12 Nutritional Biomarker Laboratory, MRC Epidemiology Unit, University of Cambridge; Cambridge, United Kingdom; 13 Lao Tropical and Public Health Institute, Ministry of Health, Vientiane, Lao People's Democratic Republic; 14 ICF, DHS Program, Rockville, MD, United States; 15 Nutrition Unit, UNICEF Uganda Coutry Office, Kampala, Uganda; 16 Caring Futures Institute, College of Nursing and Health Sciences, Flinders University, Adelaide, Australia; 17 SAHMRI Women and Kids, South Australia Health and Medical Research Institute, Adelaide, Australia

**Keywords:** vitamin B2 intake, optimal nutrition, low micronutrient status, prevalence, women

## Abstract

**Background:**

Riboflavin, as flavin mononucleotide and flavin adenine dinucleotide, is essential for numerous metabolic pathways. However, the prevalence of riboflavin deficiency worldwide remains unclear, because status biomarkers are very rarely measured in human studies.

**Objectives:**

This study aimed to investigate riboflavin status in females of reproductive age and children from several regions of the world, representing both high-income countries and low/middle-income countries (HICs and LMICs).

**Methods:**

We measured riboflavin status in population-representative samples from Ireland, United Kingdom, Cambodia, and Democratic Republic of the Congo, and in cohort samples from HICs (Northern Ireland, Spain, Canada) and LMICs (Malaysia, Lao People’s Democratic Republic, Cambodia, Uganda) using the functional assay, erythrocyte glutathione reductase activation coefficient (EGRac), with higher values indicating lower status and EGRac ≥ 1.40 indicative of deficiency.

**Results:**

In Irish (*n =* 251) and British (*n =* 163) populations, among unsupplemented females of 18–45 y, median (95% confidence interval) EGRac values were 1.39 (1.36, 1.42) and 1.40 (1.36, 1.49), and 48% and 50%, respectively, had riboflavin deficiency. In Irish females, biomarker status declined progressively(*P* < 0.002) with decreasing quintiles of dietary riboflavin intakes, from >2.1 in Q1 to <1.1 mg/d in Q5.. Females in LMICs had much higher rates of riboflavin deficiency: Malaysia (72%); Cambodia (82%); and Uganda (90%). In British children (*n =* 307), riboflavin status declined markedly with age, with median EGRac values of 1.25 (1.20, 1.28) at age 1–5 y compared with 1.40 (1.35, 1.44) at 15–17 y. In children from LMICs, 39%–75% had riboflavin deficiency, and in Ugandan children aged 5–17 y, median EGRac was 1.77 (1.39, 2.15), corresponding with clinical deficiency signs observed in this cohort.

**Conclusions:**

Riboflavin deficiency is highly prevalent in females and children across many regions worldwide. Given the wide-ranging adverse health consequences of deficiency, population-based strategies to improve riboflavin status in both LMICs and HICs are urgently needed.

## Introduction

Riboflavin (vitamin B2) is naturally found in the diet in richest supply in milk and dairy products, and to a lesser extent, in meat and eggs. It is a precursor for the cofactors FMN and FAD, which participate in intermediary metabolism by acting as proton carriers in numerous oxidation–reduction reactions, in the mitochondrial electron transport chain, central to energy production and, in conjunction with cytochrome P450, in the metabolism of drugs and toxins [1].

Of note, as FMN and FAD, riboflavin interacts with other nutrients, including iron, niacin, folate, and vitamin B6. It plays a key role in iron metabolism and in mobilizing stored iron from ferritin [2,3], and riboflavin supplementation can enhance the hematological response compared with supplementation with iron only [4], potentially contributing to preventing anemia, particularly in females of reproductive age [5,6]. Riboflavin is also essential in folate and 1-carbon metabolism, where, as FAD, it is required for the activity of methylenetetrahydrofolate reductase (MTHFR) [7,8]. The common 677C→T polymorphism in the *MTHFR* gene is associated with higher blood pressure, independently of homocysteine [[9], [10], [11]], with an estimated increased risk of hypertension by 87%, and stroke by 40%, in individuals with the *MTHFR* 677TT genotype [10]. This variant genotype results in an enzyme with decreased affinity for FAD at its active site, leading to reduced MTHFR activity [12,13], but supplemental riboflavin can effectively lower blood pressure, specifically in adults with the variant *MTHFR* TT genotype, independently of antihypertensives [[14], [15], [16]]. Also, riboflavin (as FMN) is essential in vitamin B6 metabolism, where it is required for generating pyridoxal-5’ phosphate (PLP), the active B6 form in tissues. Thus, riboflavin is a significant determinant of PLP concentrations in human studies [[17], [18], [19]], and riboflavin supplementation of older adults not only improved riboflavin status but also increased plasma PLP [20], suggesting that riboflavin may be the limiting nutrient for maintaining B6 status [21].

Given its wide-ranging interactions, the consequences of riboflavin deficiency can reflect perturbations in the metabolism of other nutrients that depend on FAD or FMN. Also, the consequences are not confined to the classic signs of clinical riboflavin deficiency (i.e., cheilosis, angular stomatitis, glossitis, dermatitis, and severe anemia with erythroid hypoplasia), but also include manifestations of subclinical deficiency that can adversely affect functioning and health throughout the lifecycle [22]. Even if not severe enough to cause clinical deficiency signs, suboptimal status is reported in both high-income countries (HICs) and low/middle-income countries (LMICs) [17,23,24].

In the first report to provide global estimates of inadequate micronutrient intakes, dietary modelling analysis recently estimated that >4 billion people worldwide do not consume enough riboflavin [25]. The status of riboflavin worldwide however, remains unclear, primarily because human studies rarely include a riboflavin status biomarker. In a recent global review reporting micronutrient status among females and children [26], it is notable that riboflavin could not be included in the analysis, precisely because the necessary data do not exist. Riboflavin status can be assessed by direct measurement of concentrations of riboflavin, FMN, and FAD in erythrocytes or plasma, or as urinary riboflavin [27]. The gold reference standard measurement for assessing long-term status, however, is the erythrocyte glutathione reductase activation coefficient (EGRac) assay, a functional biomarker which measures FAD saturation of the erythrocyte enzyme, glutathione reductase, and determines the extent to which enzyme activity is limited by riboflavin availability [27]. The EGRac assay provides a reliable biomarker for severely deficient-to-normal riboflavin status and is sensitive to riboflavin supplementation at doses ranging from 1.0 to 5.0 mg/d, for periods of ≥4 wk [28]. EGRac is expressed as a ratio of FAD-stimulated to unstimulated enzyme activity, with a higher value indicating lower riboflavin status and a cutoff value of 1.40 widely accepted to indicate deficiency [16,27,28].

Riboflavin status data (measured by any method) are greatly lacking for most countries, and thus, riboflavin deficiency often goes undetected [21]. To investigate the prevalence of riboflavin deficiency, we measured EGRac centrally to provide an assessment of status from samples collected across 9 countries worldwide. Our objective was to assess riboflavin status in females of reproductive age and children from international cohorts representing both HICs and LMICs.

## Methods

### Cohorts

The cohorts investigated include 3567 adult and child participants in 17 cohorts from 9 countries: 4 HICs in Europe and North America, and 5 LMICs in Asia and Africa. Ethical approval was sought and granted from the relevant committee for all primary cohorts except that from Uganda, because that work was deemed, by the Institutional Review Board of the United States Centers for Disease Control, to be public health and not research. Written informed consent was obtained from all adults, and from the parents/guardians of all children, in accordance with the Helsinki Declaration.

#### Population-representative cohorts

The Irish adults included in the current analysis were a subset from the National Adult Nutrition Survey (NANS), a nationally representative sample of 1500 free-living males and females, neither pregnant nor lactating. Detailed dietary, biomarker, and health and lifestyle data were collected in 20 areas covering the Republic of Ireland from 2008 to 2010. The United Kingdom (UK) National Diet and Nutrition Survey (NDNS) rolling program is a national, continuous cross-sectional survey of the diet, nutrient intake, and nutritional status of the UK general population aged ≥1.5 y, living in private households. The current analysis includes data obtained from the UK Data Service (https://www.ukdataservice.ac.uk/) for a subset of adult and child participants in years 7 (2014/15), 8 (2015/16) and 9 (2016/17) of the rolling program. During this period, the survey aimed to include a representative sample of 500 adults and 500 children each year. One of the Cambodian cohorts included in the current analysis was a representative sample of females of reproductive age in rural Prey Veng and urban Phnom Penh provinces [29]. From a Ministry of Planning list, 32 villages were randomly selected, and in each, 10 females were drawn at random from the village register. The cohort from the Democratic Republic of the Congo (DRC) were mother–child pairs participating in a cross-sectional study of the prevalence of anemia and micronutrient deficiencies, which covered 15 areas in two very different provinces: high-altitude South Kivu, and low-lying Kongo Central, along the Congo River [30]. A three-stage sampling method was used: health areas were selected using probability proportional to size, followed by random village selection. Households were chosen using a “random walk” method, and eligible participants were selected accordingly.

#### Non-population representative cohorts

Three Canadian cohorts were included in the current analysis. The first was drawn from the British Columbia Generations Project, an ongoing, prospective, research databank and biobank which recruited females from 2009 to 2013, mostly in highly populated regions of British Columbia [31]. Two other groups of Canadian females were recruited in urban Vancouver in 2013 and 2017, as described elsewhere [24,29]. The Northern Irish children included here were offspring of participants in the 2005–2006 Folic Acid Supplementation in the Second and Third Trimester (FASSTT) trial, in which healthy pregnant females were recruited at antenatal clinics in Causeway Hospital, Coleraine, Northern Ireland to receive either folic acid or placebo [32]. The follow-up FASSTT Offspring study recruited those children, at age 11 y, who agreed to give blood samples for B-vitamin biomarker analysis [33]. The Spanish infants were a subgroup of participants in the Reus and Tarragona Birth Cohort study. This is a longitudinal observational study of the gene–environment interactions associated with early life development during pregnancy and childhood, which followed up pregnant mothers recruited from 2005 to 2020 at antenatal clinics in the university hospitals Sant Joan Reus and Joan XXIII Tarragona [34].

The Malaysian participants were apparently healthy females from Kuala Lumpur, taking part in a study of riboflavin biomarker status and hemoglobin concentration [24]. One Cambodian cohort was drawn from an enhanced homestead food production trial, which used a stratified cluster design to select females from 90 farming communities in 4 districts of Prey Veng, one of the poorest provinces in Cambodia [35,36]. Another Cambodian cohort consisted of females from 26 villages in Kampong Chhnang province, who were apparently healthy but screened as anemic (capillary blood hemoglobin concentrations <117 g/L). They participated in a micronutrient intervention [37]; only preintervention data are reported here. In Uganda, UNICEF initiated an investigation into a suspected riboflavin deficiency outbreak after polio immunization workers in the Karamoja region reported a local increase in angular stomatitis and gum ulcerations after several years of severe and adverse climatic events had caused extensive crop failure and disease that devastated livestock and crops [38]. Blood samples were collected at 3 health facilities from a convenience cohort that included adults and children, both symptomatic and asymptomatic [39]. The Laotian children were a random subset of participants in the 2015–2017 Lao Zinc Study, which compared several regimens for delivering zinc to young children, both for the prevention of zinc deficiency and the treatment of diarrhea, in rural communities of Khammouane Province [40,41]. Where any study involved an intervention with micronutrients, only presupplementation data are included here.

Globally, several of the studies were carried out in regions where there is a high prevalence of genetic disorders, some of which may impact certain biomarkers, including EGRac [42]. Malaysian and Vancouver participants who at screening reported glucose-6-phosphate dehydrogenase (G6PD) deficiency, or the hemoglobinopathy *β*-thalassemia, were excluded from the study. In two of the Cambodian cohorts, and in the Laotian cohort [43], the presence of hemoglobin-E (sickle cell trait) and/or *α*-thalassemia was not an exclusion criterion, but affected individuals were identified by genotyping. In our other cohorts, genetic data were not available. We used the STROBE-nut checklist when writing our report [44].

### Sample preparation

Venous blood samples were collected from participants in 13 studies in 9 countries. In all cases, blood samples were mixed with anticoagulant (EDTA) and chilled until processed (<6 h). Plasma and erythrocytes were separated by centrifugation at ∼1800 × *g* (relative centrifugal force). After removing the plasma and buffy layer (leukocytes), the plasma was replaced with phosphate-buffered saline (pH 7.4), and samples were gently mixed and recentrifuged. This washing step was repeated twice more to remove leukocytes. EGRac has long been performed on hemolysates of thrice-washed erythrocytes because of the potential for riboflavin originating from leukocytes to affect the enzyme activity. Erythrocytes from the BC Generations Project, however, were isolated and frozen without washing. Packed erythrocyte samples were frozen and shipped on dry ice for laboratory analysis.

### Laboratory analyses

The EGRac assay was used to provide a functional assessment of riboflavin status by measuring the activity of the FAD-dependent enzyme, glutathione reductase, in hemolysates of washed erythrocytes, with and without in vitro stimulation by incubation with its cofactor FAD. The EGR activation coefficient was calculated as the ratio of FAD-stimulated to FAD-unstimulated enzyme activity as previously described [27]. Samples collected for the UK NDNS were analyzed at Medical Research Council Human Nutrition Research, Elsie Widdowson Laboratory, Cambridge, where they were assayed on microplates and read on a Thermo iEMS plate reader [45]. All other samples were batch-analyzed in duplicate, centrally at Ulster University, Coleraine, Northern Ireland, using an iLab 650 automated clinical chemistry analyzer (Instrumentation Laboratories) on samples analyzed pre-2014, and thereafter the Daytona+ (Randox Laboratories). In all cases, received samples were immediately stored appropriately and analyzed within 6 mo of receipt.

Quality control materials were frozen aliquots of lysed, washed erythrocytes with characterized EGRac values corresponding to adequate and deficient status; the mean coefficient of variation for the quality control samples was <4.0% at both laboratories. The comparability of the 2 laboratories’ methodologies was confirmed using a mean-differences analysis, performed by test-retesting of anonymous samples shipped on dry ice between the 2 centers. The systematic difference between methods was 0.01 for samples of mean EGRac value <1.40, and 0.03 for EGRac values ≥1.40, with the plate reader method producing the higher EGRac value. In addition, the random difference, calculated as 1.96 standard deviations from the mean, is within 0.20 for EGRac values <1.40, and within 0.17 for values ≥1.40.

### Dietary assessment

In NANS and NDNS, food and beverage intake data were recorded using a 4-consecutive-day semiweighed food diary, which included ≥1 weekend day. Participants were asked to record the type and amount of all food and beverages consumed and, where applicable, to record recipes, cooking method, and details of leftover food. NDNS participants were regarded as food supplement users if they self-reported use of any type of food supplement. Food portion sizes were estimated via food photographs and/or household measures. Full sampling and methodological details for NANS and NDNS have been reported previously [46,47].

### Statistical analysis

Dietary reference values used to evaluate the intakes include: average requirement (AR) 1.3 mg/d, the amount sufficient to meet the needs of half of all healthy adults; recommended daily allowance (RDA) 1.1 mg/d for females and 1.3 mg/d for males, the intake level considered sufficient to meet the needs of nearly all healthy individuals; and population reference intake (PRI) 1.6 mg/d, the intake level likely to meet the needs of almost all healthy people in a population [48]. IBM Statistics Package for the Social Sciences V28 was used for statistical analysis. Proportions of males compared with females with intake below each reference value, and proportions of males compared with females with deficient biomarker status of riboflavin, were compared using χ^2^ tests (*P* < 0.05, significant). Riboflavin intake and biomarker status were compared between groups using Mann–Whitney U tests because both reported intake data and measured EGRac data are routinely skewed.

## Results

Table 1 provides details of the study cohorts, including females of reproductive age and children in 17 cohorts from 9 countries. Table 2 shows riboflavin intake and status data for Irish and British females aged 18–45 y from population-based nutritional surveys, and for comparison, Irish and British males. Within both Irish and UK populations, females had significantly lower dietary intakes and poorer status of riboflavin than males. In Irish females, reported riboflavin intakes were 1.6 mg/d compared with 2.4 mg/d in males; and a higher proportion of females than males (34% compared with 9%) reported dietary intakes below the AR. When users of dietary supplements containing riboflavin were excluded from the analysis, daily riboflavin intakes and status were lower in both sexes, but remained significantly worse in females than in males. Among unsupplemented adults, 48% of Irish females, compared with 40% of males, had riboflavin deficiency (EGRac ≥ 1.40). In the UK population, similar status and rates of riboflavin deficiency were found.

**TABLE 1:** Description of the study cohorts.

	Study	Description	Characteristics^1^
Population-representative studies			
High income countries (adults)			
Ireland (2008–2010)	National Adult Nutrition Survey	303 females and 332 males aged 18–45 y	Living in private households in Republic of Ireland; neither pregnant nor lactating
United Kingdom (2014–2017)	National Diet and Nutrition Survey^2^	227 females and 162 males aged 18–45 y	Living in private households in the United Kingdom; neither pregnant nor lactating
High income countries (children)			
United Kingdom NDNS (2014–2017)	National Diet and Nutrition Survey^2^	386 children,1–17 y, of whom: 64 children,1–5 y; 115 children, 6–10 y; 109 children, 11–14 y; 98 children, 15–17 y	Healthy children
Low/middle income countries (adults)			
Phnom Penh/Prey Veng (2013)	Thiamin and riboflavin status in females of childbearing age in rural and urban Cambodia	302 females, 20–45 y	Healthy, neither pregnant, lactating nor taking B vitamin-containing supplements. Khmer ethnicity
Democratic Republic of the Congo (2013)	Kongo Central and South Kivu	106 females, 15–49 y	Apparently healthy, nonpregnant
Low/middle income countries (children)			
Democratic republic of the Congo (2013)	Kongo Central and South Kivu	103 children, 6–59 mo	
Non-population based studies			
High income countries (adults)			
Canada			
British Columbia (2009–2013)	British Columbia Generations Project	73 females, 35–45 y	Apparently healthy, not pregnant, ethnicity 72% European, 22% Asian, not taking B vitamin-containing supplements
Urban Vancouver (2013)		49 females, 20–45 y	Healthy, neither pregnant, lactating nor taking riboflavin-containing supplements. European and Chinese ethnicity. Self-reported *β*-thalassemia and glucose 6-phosphate dehydrogenase deficiency were excluded
Urban Vancouver (2017)		206 females, 19–45 y	
High income countries (children)			
Northern Ireland (2017)	Folic Acid Supplementation in the Second and Third Trimester Offspring Study	33 children,11 y	Children born to healthy mothers
Spain (2018)	Reus Tarragona Birth Cohort	117 children, 7.5 y	Children from a prospective pregnancy-mid childhood cohort
Low/middle income countries (adults)			
Malaysia (2017)		210 females, 19–45 y	Apparently healthy, neither pregnant, lactating nor taking riboflavin-containing supplements. Malay and Chinese ethnicity. Self-reported *β*-thalassemia and glucose 6-phosphate dehydrogenase deficiency were excluded
Cambodia			
Prey Veng (2012)	Fish On Farms Enhanced Homestead Food Production Trial	397 females,18–48 y	Neither pregnant nor taking vitamin B-containing supplements
Kampong Chhnang province (2015)	Iron with or without multiple micronutrients trial (baseline data)	262 females, 18–45 y	Apparently healthy, but hemoglobin concentration <117 g/L (by HemoCue); not pregnant. Khmer ethnicity
Uganda (2009)	Suspected riboflavin deficiency outbreak	22 females, 18–49 y	77% were symptomatic for B-vitamin deficiency
Low-middle income countries, children			
Lao People’s Democratic Republic (2016)	Lao Zinc study	261 children, 6–23 mo	No chronic medical condition, no severe illness, anemia or wasting
Uganda (2009)	UNICEF response to a deficiency outbreak in Karamoja region	16 children, 5–17 y	87% were symptomatic for B-vitamin deficiency

1 Characteristics for each cohort were identified at the time of sampling, with consideration of specific inclusion and exclusion criteria.

2 Adults and children from the National Diet and Nutrition Survey; combined data from years 7, 8, and 9 of the rolling program (2014–2015, 2015–2016, and 2016–2017, respectively).

**TABLE 2:** Riboflavin intakes and status in Irish and United Kingdom females aged 18–45 y compared with males.

Ireland [NANS^1^ (2008–2010)]	All			Users of supplements containing B-vitamins excluded		
	Males	Females	*P*	Males	Females	*P*
	*n* = 332	*n* = 303		*n* = 282	*n* = 251	
Riboflavin intake						
Median (95% CI) (mg/d)	2.4 (2.3, 2.6)	1.6 (1.5, 1.7)	<0.001	2.2 (2.1, 2.4)	1.5 (1.3, 1.6)	<0.001
Proportion below AR^2^ [*n* (%)]	29 (9)	103 (34)	<0.001	29 (10)	101 (40)	<0.001
Biomarker status (EGRac)^3^						
Median (95% CI)	1.34 (1.32, 1.36)	1.37 (1.35, 1.40)	0.006	1.35 (1.33, 1.38)	1.39 (1.36, 1.42)	0.028
Proportion ≥ 1.40 (deficient) [*n* (%)]	120 (36)	136 (45)	0.025	112 (40)	122 (48)	0.039
United Kingdom, NDNS^4^ (2014–2017)	*n* = 162	*n* = 227		*n* = 130	*n* = 163	
Riboflavin intake						
Median (95% CI) (mg/d)	1.7 (1.5, 1.8)	1.4 (1.3, 1.6)	0.003	1.5 (1.4, 1.7)	1.3 (1.2, 1.4)	<0.001
Proportion below AR^2^ [*n* (%)]	52 (32)	99 (43)	0.031	50 (38)	82 (50)	0.057
Biomarker status (EGRac)						
Median (95% CI)	1.32 (1.29, 1.37)	1.37 (1.35, 1.40)	0.023	1.36 (1.31, 1.41)	1.40 (1.37, 1.43)	0.041
Proportion ≥1.40 (deficient) [*n* (%)]	64 (40)	103 (45)	0.294	58 (45)	82 (50)	0.395

Riboflavin intakes and status were compared between sexes using Mann–Whitney U tests. The proportions of males compared with females with intakes below AR, and proportions with riboflavin deficiency, were compared using χ^2^ tests. *P* < 0.05 was considered significant.

Abbreviations: AR, average requirement; CI, confidence interval

1 National Adult Nutrition Survey (NANS) of Irish adults, years 2008–2010.

2 Average requirement; 1.3 mg/d as set by the European Food Safety Authority (EFSA) [48].

3 Erythrocyte glutathione reductase activation coefficient; riboflavin deficiency, EGRac ≥1.40.

4 Adults from the National Diet and Nutrition Survey (NDNS) of British adults; combined data from years 7, 8, and 9 of the rolling program (2014–2015, 2015–2016, and 2016–2017, respectively).

**FIGURE 1:**
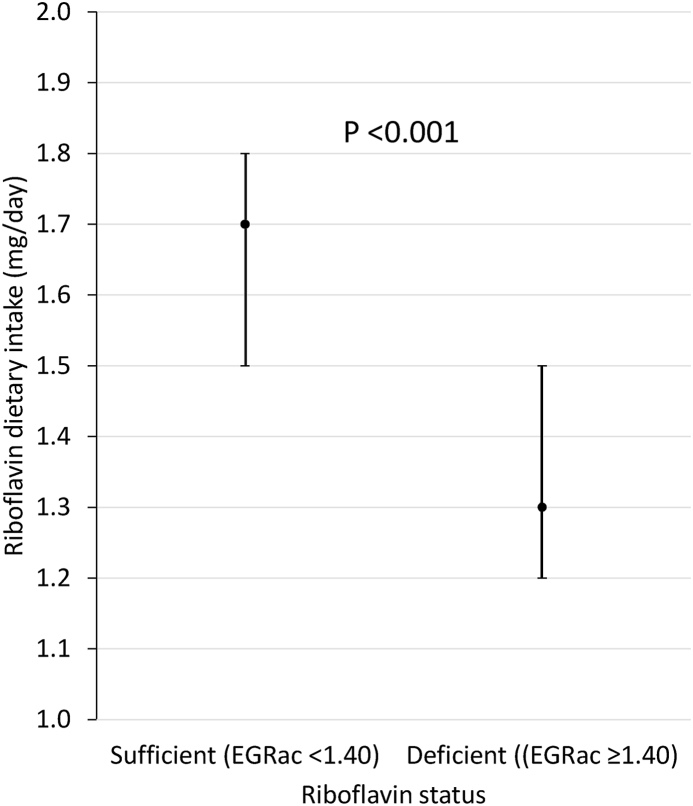
Riboflavin dietary intakes according to status in Irish females aged 18–45 y. Values are median (95% CI) riboflavin intakes mg/d. Data presented are from the National Adult Nutrition Survey (NANS) of Irish adults, years 2008–2010. Riboflavin supplement users are excluded from this analysis. CI, confidence interval; EGRac, erythrocyte glutathione reductase activation coefficient.

Table 3 shows riboflavin status (EGRac) according to quintiles of reported dietary intake in Irish females not taking riboflavin supplements. When examined by decreasing dietary intakes, corresponding status declined significantly (*P* = 0.002) in a graded relationship, from an EGRac value of 1.33 to a value of1.46, as dietary riboflavin decreased from the highest quintile (>2.1 mg/d) to the lowest quintile (<1.1 mg/d) of intakes. Figure 1 shows riboflavin intake according to status in these females. Among those identified with deficient riboflavin status (EGRac ≥ 1.40), median dietary intakes were significantly lower than in those without deficiency (1.3 compared with 1.7 mg/d; *P* < 0.001).

**TABLE 3:** Riboflavin status (EGRac) according to dietary intakes in Irish females aged 18–45 y.

Quintiles of dietary riboflavin intake					
Q1 >2.1 mg/d	Q2 1.6–2.1 mg/d	Q3 1.3–1.6 mg/d	Q4 1.1–1.3 mg/d	Q5 <1.1 mg/d	*P*^1^
*n* = 50	*n* = 50	*n* = 51	*n* = 50	*n* = 50	
1.33 (1.30,1.36)^a^	1.35 (1.30,1.40)^a^	1.44 (1.34,1.48)^ab^	1.42 (1.34,1.48)^ab^	1.46 (1.41,1.49)^b^	0.002

EGRac values are median (95% CI). Data presented are from the National Adult Nutrition Survey (NANS) of Irish adults, years 2008–2010; riboflavin supplement users are excluded.

Abbreviations: CI, confidence interval; EGRac, Erythrocyte Glutathione Reductase activation coefficient.

1 EGRac values were compared among quintiles of reported dietary intake using independent-samples medians test; *P* = 0.002. Quintiles were subsequently compared pair-wise, with Bonferroni correction for multiple testing; different superscript letters indicate significant differences between pairs.

Table 4 shows riboflavin status in females of reproductive age and in children from both HICs and LMICs. In convenience samples of Canadian females aged 18–45 y (*n* = 328), riboflavin deficiency was prevalent: 45% with an EGRac value ≥1.40. Among the 3 Canadian cohorts, median (95% confidence interval) EGRac ratios ranged from 1.37 (1.33, 1.41) to 1.40 (1.32, 1.45). In LMICs, EGRac values were generally much greater and higher rates of riboflavin deficiency were observed: Malaysia (72%); Cambodia (82%); and Uganda (90%). Among Cambodian cohorts, minimal differences in EGRac values were observed when individuals with sickle-cell disease or alpha-thalassemia were excluded. In UK children (*n =* 307), riboflavin status declined markedly with age, with median EGRac values of 1.25 (1.20, 1.28) at age 1–5 y compared with 1.40 (1.35, 1.44) at 15–17 y. In children from LMICs, 39%–75% had riboflavin deficiency, and in Ugandan children aged 5–17 y, median EGRac was 1.77 (1.39, 2.15), corresponding with clinical deficiency signs observed in this cohort.

**TABLE 4:** Riboflavin status in females and children globally.

Country	Riboflavin status (EGRac^1^)			
	Median (95% CI)	Optimal ≤1.26	Suboptimal 1.27–1.39	Deficient ≥1.40
		Number of participants (%)		
Females				
High income countries				
Canada				
British Columbia^2^ (2009–2013); (*n =* 73)	1.40 (1.32, 1.45)	16 (22)	20 (27)	37 (51)
Vancouver (2013); *n =* 49	1.37 (1.33, 1.41)	15 (31)	14 (29)	20 (41)
Vancouver, (2017); *n =* 206	1.37 (1.35, 1.40)	29 (14)	88 (43)	89 (43)
Low/middle-income countries				
Malaysia (2017); *n =* 210	1.47 (1.45, 1.50)	14 (7)	44 (21)	152 (72)
Cambodia				
All; *n =* 961	1.77 (1.73, 1.79)	66 (7)	106 (11)	789 (82)
All except severe hemoglobinopathies; *n =* 582	1.77 (1.73, 1.82)	49 (8)	52 (9)	481 (83)
Prey Veng^3^ (2012); *n =* 397	1.80 (1.76, 1.86)	27 (7)	35 (9)	335 (84)
Kampong Chhnang^4^ (2015); *n =* 262	1.75 (1.67, 1.82)	17 (6)	32 (12)	213 (81)
Cambodia Phnom Penh/Prey Veng^5^ (2013); *n =* 302	1.73 (1.66, 1.78)	22 (7)	39 (13)	241 (80)
Uganda^6^ (2009); *n =* 22	1.84 (1.59, 2.20)	1 (5)	1 (5)	20 (90)
DRC^7^ (2013); *n =* 106	1.40 (1.33, 1.47)	28 (26)	24 (23)	54 (51)
Children				
High income countries				
United Kingdom [NDNS (2014–2017)]^8^				
*n =* 47 aged 1–5 y	1.25 (1.20, 1.28)	28 (60)	10 (21)	9 (19)
*n =* 95 aged 6–10 y	1.34 (1.32, 1.36)	26 (27)	46 (48)	23 (24)
*n =* 93 aged 11–14 y	1.46 (1.37, 1.52)	17 (18)	22 (24)	54 (58)
*n =* 88 aged 15–17 y	1.40 (1.36, 1.44)	14 (16)	29 (33)	45 (51)
Northern Ireland^9^ (2017); *n =* 33 aged 11 y	1.43 (1.40, 1.51)	3 (9)	7 (21)	23 (70)
Spain^10^ (2018); *n =* 117 aged 7.5 y	1.46 (1.43, 1.51)	2 (2)	40 (34)	75 (64)
Low/middle income countries				
Lao^11^ (2016); *n =* 261 aged 6–23 mo	1.41 (1.35, 1.44)	83 (32)	42 (16)	136 (52)
Uganda^6^ (2009); *n =* 16 aged 5–17 y	1.77 (1.39, 2.15)	2 (13)	2 (13)	12 (75)
DRC (2013); *n =* 103 aged 6–59 mo	1.35 (1.27, 1.39)	40 (39)	23 (22)	40 (39)

Data are from both population-based and convenience samples, excluding users of supplements containing B-vitamins.

Abbreviations: CI, confidence interval; DRC, Democratic Republic of the Congo; EGRac, erythrocyte glutathione reductase activation coefficient; NDNS, National Diet and Nutrition Survey.

1 Erythrocyte glutathione reductase activation coefficient; a functional biomarker of riboflavin status whereby: ≤1.26 indicates optimal status; 1.27–1.39, suboptimal; ≥1.40, deficient. A value of ≥1.27 was used to indicate suboptimal status based on postintervention EGRac values measured in a previous study by our group, using low-dose riboflavin (1.6 mg/d) for 16 wk [[14], [15], [16]].

2 British Columbia Generations Project.

3 Fish on farms enhanced homestead food production trial.

4 Iron with or without multiple micronutrients trial (randomized controlled trial, baseline data).

5 Determination of thiamin and riboflavin status in females of childbearing age in rural and urban Cambodia.

6 UNICEF response to a deficiency outbreak in Karamoja region.

7 Democratic Republic of the Congo (Kongo Central and South Kivu).

8 National Diet and Nutrition Survey, data for children aged 1–5 y. For every age-group, data are combined from y 7, 8, and 9 of the rolling program: 2014–2015, 2015–2016, and 2016–2017, respectively.

9 Folic acid supplementation in the second and third trimester offspring study.

10 Reus Tarragona Birth Cohort.

11 Lao Zinc Study.

**FIGURE 2:**
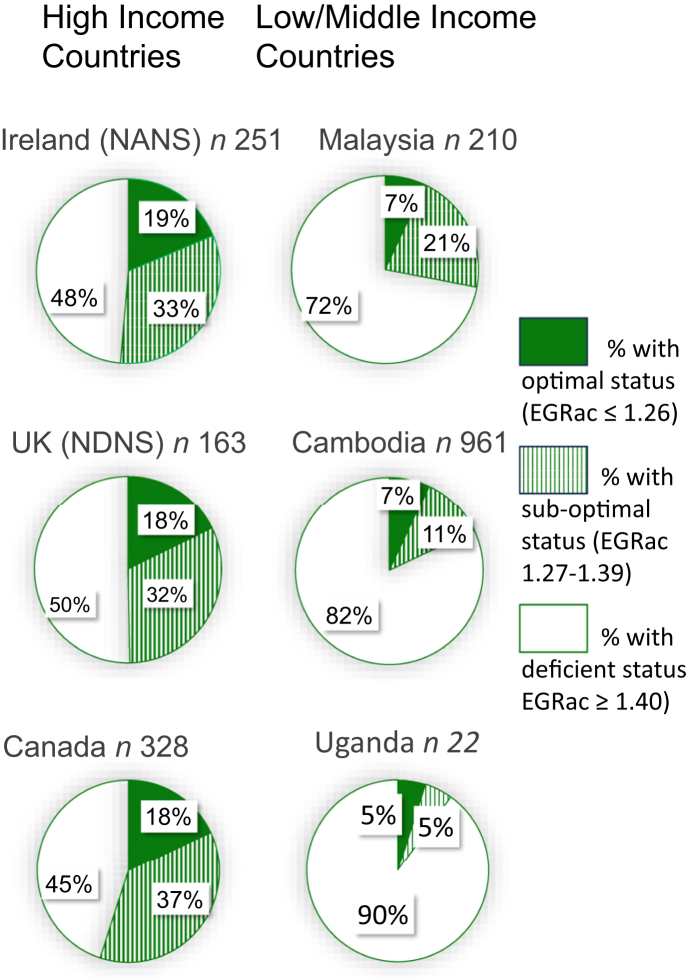
Riboflavin status in females of reproductive age from 6 countries worldwide. Riboflavin status was defined using the erythrocyte glutathione reductase activation (EGRac) coefficient. Pie charts show percentage with riboflavin deficiency (EGRac ≥1.40), suboptimal status (1.27–1.39), and optimal status (≤1.26). A value of ≥1.27 was used to indicate suboptimal status based on postintervention EGRac values measured in a previous trial of low-dose riboflavin supplementation (1.6 mg/d) for 16 wk [16]. Data exclude pregnant females and users of dietary supplements containing B-vitamins. High Income Countries: Ireland (population-based cohort, National Adult Nutrition Survey); United Kingdom (population-based cohort, National Diet and Nutrition Survey); Canada (apparently healthy females in British Columbia and urban Vancouver). Low/middle-Income Countries: Malaysia (apparently healthy females); Cambodia (apparently healthy females in Phnom Penh and Prey Veng, and females in Kampong Chhnang with hemoglobin concentration <117 g/L); Uganda (Karamoja females with and without symptoms of clinical riboflavin deficiency). NANS, National Adult Nutrition Survey; NDNS, National Diet and Nutrition Survey.

Figure 2 shows riboflavin status in females of reproductive age from 6 countries worldwide. These data show that although riboflavin deficiency in females was most pronounced in LMICs (72%–90% in Malaysia, Cambodia, and Uganda), it was also evident in females from HICs, ranging from 45% to 50% in Canada, Ireland, and the UK.

## Discussion

This study, investigating riboflavin status in 17 cohorts from 9 countries, both HICs and LMICs, shows that riboflavin deficiency is highly prevalent in females of reproductive age, and children. Although riboflavin deficiency was most pronounced in LMICs, it was also evident in females from HICs. In countries providing samples from children, riboflavin status was found to decline with age during childhood, and riboflavin deficiency affected over half of children in the UK by age 11–17 y. In Ugandan children, riboflavin deficiency was evident in the majority of 5–17-y-olds sampled, corresponding with clinical deficiency signs observed in this cohort.

In population-representative cohorts, high proportions of females of reproductive age had low dietary intakes of riboflavin, and correspondingly, status was generally low, with a high prevalence of riboflavin deficiency identified. These findings provide important reference data for riboflavin status, considering that human studies rarely include a riboflavin biomarker and it is absent from the vast majority of population-based nutrition surveys, including those from the United States and Canada. In LMICs, we observed particularly high rates of riboflavin deficiency in females of reproductive age. The highest rate of deficiency was found in Uganda (Karamoja region), where the United Nations World Food Programme had previously launched a humanitarian response with a general food distribution intended to replace 70% of the food supply. Because the putative cases of riboflavin deficiency investigated in the current report were based on observations of classical clinical symptoms (namely, angular stomatitis, and gum ulcerations), blood samples were collected from both symptomatic and asymptomatic cases [39]. Riboflavin deficiency was highly prevalent in both categories, perhaps not surprisingly, given that such symptoms are rather nonspecific and can also occur with other micronutrient deficiencies. Furthermore, analysis of the food distributions by the UN later revealed that the mean riboflavin content was only 0.23–1.6 mg/d [39]. Among the LMICs countries in the current study, we detected the lowest rate of riboflavin deficiency in DRC and found EGRac values that appeared similar to those of Canadian females. The likely explanation for this unexpected finding is that the measured EGRac values in this case may have been a measurement artifact, rather than accurately reflecting riboflavin status. G6PD deficiency and *β*-thalassemia are common in sub-Saharan Africa [27,49] and are associated with disrupted erythrocyte flavin metabolism that can generate misleading EGRac test results; for example, in individuals with G6PD deficiency, EGRac values within the normal range can be found even in the presence of clinical signs of riboflavin deficiency [50].

The greater rates of riboflavin deficiency in LMICs compared with HICs, as revealed by the current EGRac analysis, are likely related to much lower dietary intakes in these countries. Although dietary intakes are not reported here for LMICs, an estimated 4 billion people worldwide (55% of the global population) do not consume enough riboflavin, with greater dietary inadequacy in females compared with males, and Africa is identified as a region where females are at particular risk [25]. Median riboflavin intakes as low as 0.7 mg/d have been reported in Congolese females [51], whereas in children aged 1–3 y, riboflavin intake was reported as 0.6–0.7 mg/d [52]. Other findings from sub-Saharan Africa include reported mean riboflavin intakes of 0.9 mg in Tanzanian adults [53], whereas in Mali, in West Africa, reported intakes were 0.7 mg/d [54]. Such low dietary intakes are of particular concern given that clinical signs of riboflavin deficiency in humans appear at intakes of <0.5–0.6 mg/d [55] and population-wide dietary insufficiency is strongly associated with new-onset hypertension [56]. Milk and dairy products are the richest dietary sources of riboflavin, and therefore countries that have low intakes of this food group will be at greatest risk of riboflavin deficiency [22]. In China, where dairy foods are rarely consumed, resulting in extremely low dietary riboflavin as reported in population-wide survey data, riboflavin insufficiency is widespread [56,57]. Certain countries, predominantly in North and South America, have mandatory riboflavin enrichment policies [22], but unlike fortification, this serves only to replace the riboflavin lost during milling of grain [22], and the levels of added riboflavin are typically low. Nonetheless, this will benefit riboflavin intakes to some extent when compared with other countries without this policy. However, in the absence of biomarker data in most countries, the adequacy of riboflavin nutrition globally remains uncertain. The lack of biomarker data (with reliance only on reported dietary intakes) is especially a concern for critical life stages, particularly pregnancy, where riboflavin requirements are elevated [48].

In the current study, riboflavin intakes in Ireland and the UK were broadly in line with available population-representative dietary data from other HICs [[58], [59], [60]]. Between 1999 and 2018, the United States NHANES, the British Columbia Nutrition Survey, and the Canadian Community Health Survey have all reported mean daily riboflavin intakes in the range of 1.5–1.8 mg for females, and 2.1–2.8 mg for all adults [[61], [62], [63], [64]]. Although riboflavin biomarkers are not currently reported in NHANES, in a recent study of intakes, drawing on NHANES data in >10,480 United States adults, dietary riboflavin was found to be inversely associated with all-cause and cardiovascular disease mortality [65]. However, interpreting dietary data across countries is complicated by the fact that different methodologies have been used. Dietary recommendations can also vary considerably worldwide, with the United States RDA value set at 1.1 mg/d for females [55], whereas the much more recent European PRI was set at 1.6 mg/d [48]. In Irish females in this study, reported riboflavin intakes were 1.6 mg/d compared with 2.4 mg/d in males,and a much higher proportion of females than males (34% compared with 9%) reported dietary intakes below the AR. Likewise, in North American survey data, dietary intakes are consistently reported to be lower in females than in males [61,63,64], and median intakes ranging 1.4–1.7 mg/d have been reported for Dutch and Japanese females, respectively [66,67]. Notably, mean dietary riboflavin intakes as low as 0.8 mg/d were reported in adults in The China Health and Nutrition Survey [56].

Among population-representative nutrition surveys, very few have measured riboflavin biomarker status, relying instead on dietary intake data only [22]. Thus, the body of published data on riboflavin biomarkers is limited and characterized by considerable variation among study methodologies. These include choice of assay, inconsistencies in cut-off points to define deficiency, the measurement of riboflavin concentration variously in blood, plasma, or urine, and the use, in supplementation trials, of multinutrient interventions, which can confound interpretation of riboflavin results [68]. The population-based data presented here, which provide riboflavin intake and corresponding status in the same individuals from Ireland and the UK, address this gap to some extent and make it possible to consider riboflavin status in relation to dietary intakes, and also dietary riboflavin in females above and below the cutoff point for biomarker deficiency. When riboflavin status in Irish females was examined according to dietary intakes, from the highest to lowest quintile, we found a graded decline in status with decreasing dietary riboflavin. Furthermore, in females with deficient riboflavin status (EGRac ≥ 1.40), corresponding dietary intakes were significantly lower than in those without biochemical deficiency. This provides good evidence of the validity of EGRac as a reliable biomarker reflective of intakes across the dietary range, as we previously concluded from a systematic review of biomarker responses to intervention with riboflavin [28].

Rather uniquely, we report data on riboflavin status in children from 5 countries, both HICs and LMICs. Riboflavin deficiency was found to affect three-quarters of 5–17-y-olds in Uganda, consistent with the clinical deficiency signs observed in this cohort. In the UK, riboflavin status showed a decline with age from young children to adolescents, and although EGRac values at 1.5–5 y were well within the normal range, riboflavin deficiency affected over half of 11–17-y-olds. The observation of worse riboflavin status in older compared with younger children was previously reported by us, and we showed that the progressive increase in homocysteine concentrations from 4- to 18-y-olds reflected decreases in the status of the 4 metabolically interrelated B vitamins, namely folate, vitamin B12, B6, and riboflavin [69]. Likewise, in non-population based cohorts in the current study, riboflavin deficiency was more evident in the older children. Low status of riboflavin in childhood is likely to have lasting impacts on health. Also, among Laotian children, those deficient in riboflavin were more likely to be wasted and to have anemia, whereas other measured nutrients did not show similar associations [41]. The association of riboflavin deficiency with anemia, related to riboflavin’s essential role in iron metabolism and status, is largely overlooked in research and public health settings. With an estimated 47% of preschool children worldwide affected by iron deficiency anemia [70], which is primarily the result of inadequate iron intake, more investigation is needed on the potential role of riboflavin intervention in helping to alleviate childhood anemia [71,72].

Considering the diverse roles of FAD- and FMN-dependent enzymes, riboflavin deficiency could contribute to a range of adverse health outcomes across the life course, including anemia [72]. Notably, the addition of riboflavin to iron supplements was shown to improve the hematologic response to treatment in anemic, riboflavin-deficient young females in the UK [73].whereasLikewise, in anemic pregnant Chinese females, riboflavin significantly improved the response to treatment compared with iron and folic acid alone [74]. Riboflavin deficiency can also contribute to stroke and to hypertension [75], and this is particularly evident in populations with low riboflavin intakes, such as China [56], and in subpopulations worldwide who are genetically at risk of hypertension [10,11], where targeted riboflavin intervention is proven to effectively lower blood pressure [[14], [15], [16]]. Finally, deficient riboflavin intake can adversely affect vitamin B6 status given its metabolic dependency on FMN and the proven role of riboflavin in determining PLP concentrations in human studies [17,20], with potentially important health consequences, considering that low PLP is associated with increased risk of cancer, cardiovascular and neuropsychiatric diseases [76,77], and all-cause mortality [23].

A notable strength of the current study is that we investigated riboflavin status in females of reproductive age and children from several regions of the world, representing both HICs and LMICs. For all analyses, we used the gold-standard biomarker, EGRac, and all the samples were analyzed centrally in one laboratory (Ulster University), apart from the NDNS samples, which were analyzed in the University of Cambridge, UK. Both laboratories routinely perform EGRac analysis and share samples in assay validation studies for quality assurance purposes, with generally excellent agreement between our centers. The study was, however, not without limitations. Some of the cohorts included were drawn from regions where certain genetic conditions known to affect erythrocyte flavin metabolism are common, potentially resulting in misleading EGRac test results [50]. This is particularly the case in sub-Saharan African populations, where G6PD deficiency affects an estimated 7.5% of people in malaria-endemic areas [49], which may have impacted our results to some extent in the Congolese cohorts. Of note, however, in the Malaysia and Vancouver cohorts, individuals with G6PD deficiency and *β*-thalassemia were excluded.In two Cambodian cohorts, individuals with hemoglobin E (sickle cell trait) and α-thalassemia, the most severe and common hemoglobinopathies in the region, were identified but included in the data, making it possible to re-examine the Cambodian data after excluding such affected individuals. Because neither the EGRac values nor the proportions with riboflavin deficiency were altered, it seems unlikely that the inclusion of individuals with these two conditions significantly impacted our overall results. Although the inclusion of non-population representative samples in 5 of the 9 countries in this study may be considered as a limitation, this is reflective of the general paucity of riboflavin status data from human studies worldwide. Notably, we included data from the only 2 countries globally to provide a riboflavin status measure as part of their rolling national nutrition surveys, namely the UK and Ireland.

In conclusion, the current findings reveal that riboflavin deficiency is highly prevalent in females and children across many regions worldwide, with those in LMICs at greatest risk. Unlike previous studies that have typically relied on dietary riboflavin intake data only, the current study draws on 2 population-based cohorts (from Ireland and the UK) to link dietary intakes with corresponding riboflavin biomarker values, and the results show that females are at particular risk both of dietary insufficiency and low/deficient status. Given the adverse health consequences of deficiency, population-based strategies to improve riboflavin status in both LMICs and HICs are needed. This study is a first step in providing evidence to underpin emerging dietary recommendations and food policy measures concerning riboflavin. Further research, in the form of randomized trials within the dietary range of riboflavin intakes, should investigate the effects of interventions aimed at improving riboflavin status and preventing the clinical sequelae of riboflavin deficiency.

## Author contributions

The authors’ responsibilities were as follows – HM, MW, LM: designed the research; LM, AM, RB, HRJ, LH, CH, KP, PB, CDK, KCW, DAP, TM, G-MH, NB: conducted research (hands-on conduct of the experiments and data collection); LM, HM, MW, HRJ, MAK: analyzed the data and performed the statistical analysis; LM, HM, MW: wrote the paper and had primary responsibility for the final content; and all authors: provided critical review, contributed to the manuscript development, and read and approved the final manuscript.

## Data availability

Data described in the manuscript, codebook, and analytic code will be made available upon request pending application and approval.

## Funding

The study received funding from UKRI–Biotechnology and Biological Sciences Research Council (BBSRC); Grant award No BB/P028241/1 (The DERiVE project; awarded to MW) under the international Joint Programming Initiative ERA-HDHL call on Biomarkers for Nutrition and Health. The funders had no role in the design, methods, subject recruitment, data collection, analyses or preparation of this paper. DAP and TM are supported by the NIH and Care Research (NIHR) Cambridge Biomedical Research Centre (NIHR203312).

## Conflict of interest

MW reports financial support was provided by UKRI Biotechnology and Biological Sciences Research Council (BBSRC). MW, HM, and JJS have patent # EP2139488 (now expired); issued to none. CDK is an Editorial Board Member for *The Journal of Nutrition*. Given her role as Editorial Board Member, she had no involvement in the peer review of this article and had no access to information regarding its peer review. Full responsibility for the editorial process for this article was delegated to another journal editor. If there are other authors, they declare that they have no known competing financial interests or personal relationships that could have appeared to influence the work reported in this paper.
