# Supplementary material for: Riboflavin Deficiency Is Highly Prevalent in Females and Children across High and Low/Middle Income Countries Worldwide
Source: J Nutr. 2026 Feb 23;156(3):101277. doi: 10.1016/j.tjnut.2025.101277 (PMC13014503; doi:10.1016/j.tjnut.2025.101277)
Supplement: multimedia component 1 [file mmc1.docx]

|  | **Study** | **Description** | **REC references** |
| --- | --- | --- | --- |
| Ireland  (2008–2010) | National Adult Nutrition Survey | 303 women and 332 men aged 18-45 years | Clinical Research Ethics Committee of Cork Teaching Hospitals, University College Cork Ref: ECM 3 (p) 04/11/08 |
| UK  (2014-2017) | National Diet and Nutrition Survey | 227 women and 162 men aged 18-45 years  386 children,1-17 years | Cambridge South National Research Ethics Service Committee Ref. 13/EE/0016 |
| Cambodia Phnom Penh / Prey Veng (2013) | Thiamin and riboflavin status in women of childbearing age in rural and urban Cambodia | 302 women, 20-45 years | UBC CREB^1^ Ref. H12-­02847;  Cambodia NECHR^2^ Ref. 0004-NECHR, 04/01/2013 |
| Democratic Republic of Congo (2013) | Kongo Central and South Kivu | 106 women, 15-49 years  103 children, 6-59 months | UBC CREB Ref. H14-01279; Université de Kinshasa  Ref. ESP/CE/033/14;  Université Catholique de Bukavu  Ref. UCB/CIE/NC/25/2014 |
| British Columbia (2009-2013) | British Columbia Generations Project | 73 women, 35-45 years | UBC CREB Ref. H17-01239 |
| Canada Urban Vancouver (2013) |  | 49 women, 20-45 years | UBC CREB Ref. H12­-02847 |
| Canada Urban Vancouver (2017) |  | 206 women, 19-45 years | UBC CREB Ref. H15-00521 |
| Northern Ireland (2017) | Folic Acid Supplementation in the Second and Third Trimester Offspring Study | 33 children,11 years | Ulster University Research Ethical Committee Ref. 12/0121 |
| Spain (2018) | Reus Tarragona Birth Cohort | 117 children, 7.5 years | CEIM^3^, Institut d’Investigació, Sanitària Pere Virgili. Código: PI19/00844. Ref CEIM: 022/2019 |
| Malaysia (2017) |  | 210 women, 19-45 years | UBC CREB H15-00521 |
| Cambodia Prey Veng (2012) | Fish On Farms Enhanced Homestead Food Production Trial | 397 women,18-48 years | UBC CREB H12-00451 |
| Cambodia, Kampong Chhnang province (2015) | Iron with or without multiple micronutrients trial (baseline data) | 262 women, 18-45 years | UBC CREB Ref. H15-00933; Cambodia NECHR Ref. 110-NECHR |
| Uganda (2009) | Suspected riboflavin deficiency outbreak | 22 women, 18-49 years  16 children, 5-17 years | Institutional Review Board of the US Centers for Disease Control deemed this emergency response as public health practice, not research. |
| Lao People’s Democratic Republic (2016) | Lao Zinc Study | 261 children, 6-23 months | Lao PDR^4^ Ministry of Health NECHR Ref. 40/2014, 069/2015, 039/2016; UBC Institutional Review Board Ref. 626187 |

1. University of British Columbia [Clinical Research](https://protect.checkpoint.com/v2/r02/___https:/www.sciencedirect.com/topics/pharmacology-toxicology-and-pharmaceutical-science/clinical-research___.YzJlOnVsc3RlcnVuaXZlcnNpdHk6YzpvOmJjOTNjNDY2ZDhiMTVmOGI1NzgwMjNkOTA2NDY0ZGZhOjc6YmU0Mzo3MGNhNDYyNGNiZTVmYjQzODQ5MGMzNjQyMjJiN2U3ODU1NTNmOWIzNDI5Yzk4YzAxMGJiY2MwMzEyNTcwMGYxOmg6VDpO) Ethics
2. National Ethics Committee for Health Research
3. Comitè Ètic d’Investigació amb Medicaments
4. People’s Democratic Republic
